# Sky Detection in Hazy Image

**DOI:** 10.3390/s18041060

**Published:** 2018-04-01

**Authors:** Yingchao Song, Haibo Luo, Junkai Ma, Bin Hui, Zheng Chang

**Affiliations:** 1 Shenyang Institute of Automation, Chinese Academy of Sciences, Shenyang 110016, China; luohb@sia.cn (H.L.); majunkai@sia.cn (J.M.); huibin@sia.cn (B.H.); changzheng@sia.cn (Z.C.); 2 University of Chinese Academy of Sciences, Beijing 100049, China; 3 Key Laboratory of Opto-Electronic Information Processing, Chinese Academy of Sciences, Shenyang 110016, China

**Keywords:** sky detection, sky labeling, haze-relevant features, perceptual hazy density, imbalance classifier, HazySky

## Abstract

Sky detection plays an essential role in various computer vision applications. Most existing sky detection approaches, being trained on ideal dataset, may lose efficacy when facing unfavorable conditions like the effects of weather and lighting conditions. In this paper, a novel algorithm for sky detection in hazy images is proposed from the perspective of probing the density of haze. We address the problem by an image segmentation and a region-level classification. To characterize the sky of hazy scenes, we unprecedentedly introduce several haze-relevant features that reflect the perceptual hazy density and the scene depth. Based on these features, the sky is separated by two imbalance SVM classifiers and a similarity measurement. Moreover, a sky dataset (named HazySky) with 500 annotated hazy images is built for model training and performance evaluation. To evaluate the performance of our method, we conducted extensive experiments both on our HazySky dataset and the SkyFinder dataset. The results demonstrate that our method performs better on the detection accuracy than previous methods, not only under hazy scenes, but also under other weather conditions.

## 1. Introduction

Sky is a strong indicator of outdoor images and provides information about the environment. Sky detection plays an essential role in a wide range of vision applications, such as vision-based ground robot navigation [1], obstacle avoidance for unmanned aerial vehicles [2] and unmanned surface vehicles [3], image editing [4,5], weather classification [6,7] and scene parsing [5,8,9,10]. Sky detection also facilitates some image enhancement tasks. For example, estimation of the airlight is a crucial step for image dehazing methods [11,12,13]. Accurate and effective detection of sky will contribute to the estimation of the airlight. In view of the significance of sky detection, numerous scholars have carried out extensive research on this challenging problem.

Many previous methods mainly focus on solving two problems of sky detection. The first one is distinguishing between sky and non-sky regions that are sky-like in appearance, such as blue water, gray walls and white buildings. Shen et al. [1] proposed a horizon line detection approach trying to address this problem by using gradient information. The method seeks to find a position in each column of the image and labels pixels above this position as sky, otherwise as non-sky. Another challenge that previous methods seek to solve is the partial occlusion of the sky by foreground objects cutting the sky into many disconnected parts. In this case, horizon line detection-based method [1] will lose efficacy, and classification-based approaches [6,8,9,14] may be more effective. Therefore, methods combining hand-engineered features with a classifier were used to address this problem. Lu et al. [6] extracted 128 dims SIFT features and three dims color features and trained a random forest classifier. Shang et al. [14] made use of features like texture, lines, position and shape to train an SVM classifier. Hoiem et al. [8] estimated the geometric properties of a scene and trained a greedy segmentation algorithm. Tighe et al. [9] introduced an image parsing method by performing scene-level matching and superpixel-level matching to segment image regions into different semantic classes including “sky”.

Although these method have achieved good results on their own test sets, their performance still needs to be further verified, because their experimental datasets were captured under favorable conditions that cannot reflect the variational appearance of the sky [15]. The establishment of the SkyFinder dataset [15] poses new challenges to previous methods [1,6,8,9,14]. These methods, being trained on an ideal dataset, may perform poorly when facing unfavorable conditions like the effects of weather, season and time. Mihail et al. [15] built the SkyFinder dataset and evaluated three existing state-of-the-art sky labeling methods [6,8,9] on this dataset. The results showed that the performance of these algorithms varies significantly with the change of illumination conditions and weather conditions.

To make up the shortcoming that hand-engineered features adapt poorly to the variational appearance of the sky, two more powerful sky labeling models [15,16], based on deep neural networks, were designed and tested on the SkyFinder dataset. Mihail et al. trained an rCNNmodel by adding the output of three baseline methods [6,8,9] to the training set and achieved a lower MCR (MisClassificationRate) than the three baseline methods. Place et al. [16] trained a RefineNet model on the SkyFinder dataset and achieved a lower MCR than Mihail et al. across their own testing split.

Although deep learning-based models achieved excellent performance, millions of weight optimizations, a large amount of convolution operations, the long time for training and the huge number of samples requirement greatly limit their application in practical systems. In contrast, traditional methods (hand-engineered features + classifier) are more flexible, less computing resource consuming, have easier convergence when training and fewer sample requirements, therefore being more suitable for practical applications (e.g., the embedded system application). Place et al.’s [16] experiments also demonstrated the fact that off-the-shelf models, even trained on a huge dataset, would produce poor results when the application environment changes. Therefore, existing methods are still effective so long as they have been modified to suit the task’s need.

**Figure 1:**
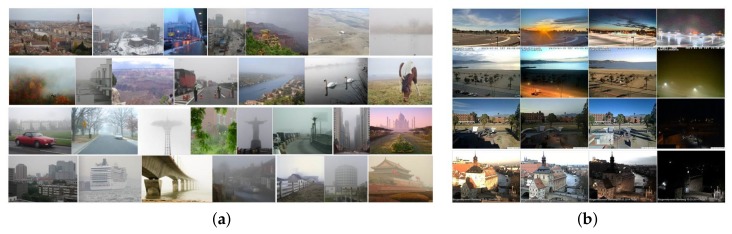
Sample images from (**a**) the HazySky dataset, and (**b**) the SkyFinder dataset.

In this paper, we pursue the challenges of sky detection in adverse weather (e.g., fog, haze and mist) and propose a novel sky detection approach from the perspective of probing the density of haze. Haze (in this paper, we do not discriminate between fog, haze or mist and express them uniformly in one term as haze) is a typical representative of bad weather. In hazy weather, atmospheric suspended particles, such as water vapor, dust and smoke, scatter the scene radiation and obscure the scene clarity. Outdoor images are often contaminated by haze, even on a sunny day. Influenced by haze, images may suffer from low contrast, faint color, blurred texture and shifted luminance. Existing hand-engineered features cannot well express distinctions between sky and some non-sky regions affected by haze. Inspired by image dehazing methods [11,13,17,18], we characterize sky by several haze-relevant features. As the presence of haze is an important cue for humans to perceive depth [11], regions with the farthest depth have the most probability of being sky. Therefore, we identify the sky by detecting the most opaque region. The contributions of our paper are summarized as follows:

- Feature extraction: We creatively introduce the haze-relevant features that reflect the perceptual hazy density and the depth of scene to characterize sky.
- Region classification: To make up for the shortages of a single classifier, we propose a multiple classifier idea that trains two imbalance SVM classifiers, a sky-concerned one and a non-sky-concerned one, to divide the image into three subregions: high confidence sky regions, high confidence non-sky regions and uncertain regions. Then, these high confidence regions are taken as the reference to further label the uncertain regions.
- Dataset setting up: To train the model and verify the performance, we build a sky dataset named HazySky (our HazySky dataset can be available at: https://pan.baidu.com/s/1c8uufSfo2pTItHzjwWbNmQ) with 500 labeled natural scene hazy images. Compared to SkyFinder, our HazySky contains more abundant scene contents and more shooting angles. Figure 1a,b shows some sample images from the two datasets.

We conduct extensive experiments both on our HazySky dataset and the SkyFinder dataset. The results indicate that our haze-relevant features based model uses fewer features and achieves better performance both on detection accuracy and on misclassification rate than previous methods under different weather and lighting conditions.

## 2. Dataset

The HazySky dataset is composed of 500 natural scene hazy images with annotated sky regions. These images cover 476 different scenes and abundant image contents, such as buildings, roads, mountains and rivers, animals and human beings. The images are not limited to sky scenes, but also scenes without sky. We mark the sky regions by image segmentation [19] and manual labeling.

These hazy images were collected from several scholars’ studies [11,12,13,17,20] about image dehazing. The first part of our dataset contains 375 hazy images picked from Choi’s [17] dataset. These images cover diverse image contents and different levels of haze density. We selected these images by following the principle that the border lines between sky and non-sky regions can be recognized by human eyes so that the human-labeled sky can be taken as the benchmark. The second part of our dataset contains 25 images picked from Zhan’s [20] dataset. These images were captured during a long time recording of the weather variations of one place in Hefei city. The last 100 images of our dataset were collected from He et al.’s, Song et al.’s and Berman et al.’s study [11,12,13]. These images often appeared in the literature of the image dehazing algorithms.

The average coverage of sky pixels in our HazySky dataset is 25.52%, with a standard deviation of 17.97%. This ratio is in line with people’s daily photographing habits, because more attention would be paid to the foreground objects rather than the sky background.

Another larger sky dataset SkyFinder [15] contains about 90K outdoor images captured by 53 static webcams over long periods of time. Each camera captured thousands of images from the same scene at different times. The average coverage of sky pixels in the SkyFinder dataset is 41.19%, with a standard deviation of 15.71%.

Compared to the SkyFinder dataset, our HazySky dataset contains more abundant scene contents and more shooting angles of hazy images, as shown in Figure 1a, while the SkyFinder pays much attention to the variational appearance of the sky impacted by weather and lighting conditions, as shown in Figure 1b. It covers only 53 different scenes, and the similarity of different scenes is a bit high.

## 3. The Proposed Approach of Sky Detection

In general, existing sky detection methods often include three essential steps. First, characteristics that are unique to sky would be identified and quantified. Second, by using these quantitative features, a suitable classification or segmentation method is employed to divide the image roughly into sky and non-sky regions. However, misclassification may occur during the classification and segmentation process. Therefore, subsequently, a post-processing step is conducted to refine the result and reduce error.

**Figure 2:**
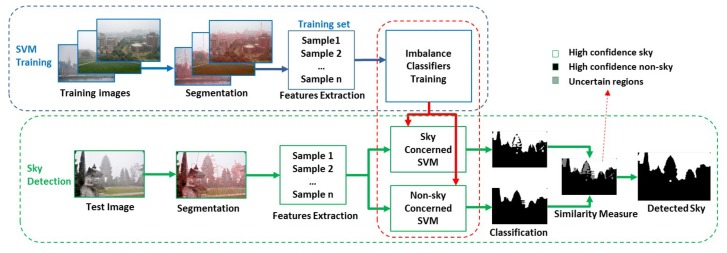
Illustration of the proposed sky detection approach.

Our approach also includes the above steps. The schematic diagram of our method is shown in Figure 2. The training process includes image segmentation, feature extraction and classifier training. This process will produce two imbalance SVM classifiers with different performance in sky and non-sky regions, respectively. In the process of sky detection, we utilize these two SVMs to divide the test image into three subregions: high confidence sky regions, high confidence non-sky regions and uncertain regions. Then, these high confidence regions are taken as the reference to label the uncertain regions.

### 3.1. Image Segmentation

For extracting region-level features, an image segmentation step is conducted. There are many excellent image segmentation methods that can be employed. To show the flexibility of our method, we employ two state-of-the-art image segmentation approaches. They are respectively the hierarchical segmentation approach [19] and the graph-based segmentation approach [21]. Both of them are the classical methods in the field of image segmentation and have been widely cited in the literature of computer vision. The graph-based method [21] has a lower time complexity, while the hierarchical segmentation method [19] produces better segmentation results. In a hazy scene, object edges become blurred. To produce fine edges on the detection results, the parameters of the two methods are all set to produce over-segmentation results (we set UCM${}_{th}$ = 0.01 when the hierarchical segmentation method [19] is used and set sigma = 0.01, k = 200, min = 20 when the graph-based method [21] is used).

### 3.2. Features Extraction

**Figure 3:**
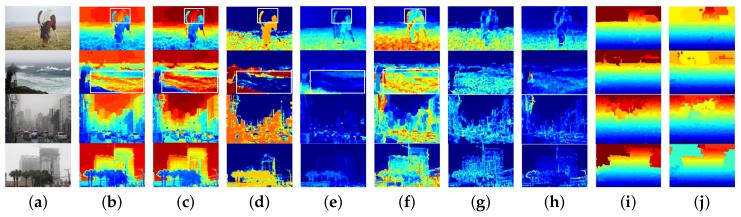
Haze-relevant features used in this paper. (**a**) Hazy images; (**b**) dark channel; (**c**) depth of scene; (**d**) hue disparity; (**e**) color saturation; (**f**) contrast energy; (**g**) Canny edge; (**h**) color gradient; (**i**) maximum height; (**j**) minimum height.

In the task of sky detection, a good feature plays an essential role in the performance of the algorithm. We believe that proper features will yield twice the result with half the effort. In this paper, we unprecedentedly introduce several haze-relevant features that reflect the density of haze and the depth of the scene to characterize sky. All features are extracted from small segmentation regions produced by the method [19,21]. Features used in this paper are shown in Figure 3a,b.

#### 3.2.1. Color Features

f1: Dark channel

The dark channel feature is a rough approximation of the density of haze. The dark channel prior [11] denotes that: in most of the non-sky regions of haze-free images, there are always some pixels (dark pixels) with very low intensity in at least one color channel (dark channel). The presence of haze causes these dark channels to no longer be dark. We use a pixel-wise dark channel to express the density of haze, as shown in Equation (1) [11].

(1) $$I_{d}\left(x\right)=min_{c\in \left\{r,g,b\right\}}I^{c}\left(x\right)$$

Here, *x* is the pixel coordinate, and *c* represents the R,G,B color channels. I(x) is the input hazy image. Region-level dark channel $I_{d}\left(s\right)$ is expressed as the average value of $I_{d}\left(x\right)$ in each segmentation region, as shown in Figure 3b, and warm colors denote sky regions or white objects, while cool colors denote foreground objects.

f2: Scene depth

To model the scene depth in a hazy image, a linear model was built based on the color attenuation prior [18]. It denotes that the brightness and the saturation of a hazy image vary sharply along with the change of the haze density. With the increase of haze density, the difference between the brightness and the saturation also increases. The scene depth in hazy images is modeled by Equation (2) [18].

(2) $$D\left(x\right)=c_{0}+c_{1}\cdot B\left(x\right)+c_{2}\cdot S\left(x\right)+\varepsilon \left(x\right)$$

Here, B(x) and S(x) are the brightness and the saturation of pixel *x*. c${}_{0}$–c${}_{2}$ are the fixed parameters learned by a supervised learning model, and ε(x) denotes the random error of the model. In this paper, they are set as c${}_{0}$ = 0.121779, c${}_{1}$ = 0.959710, c${}_{2}$ = −0.780245 and ε(x)≡0. Region-level scene depth D(s) is expressed as the average value of D(x) in each segmentation region, as shown in Figure 3c.

f3: Hue disparity

To identify regions affected by haze, Ancuti et al. [22] proposed a haze detector that computes the hue disparity between the original image and the semi-inverse image. The detector is based on the observation that the hue disparity has a small value in the sky or dense haze regions, while it has a big value in the foreground regions. The semi-inverse image $I_{si}^{c}\left(x\right)$ and hue disparity $H_{d}\left(x\right)$ are defined as Equations (3) and (4) [22].

(3) $$I_{si}^{c}\left(x\right)=max\left\{I^{c}\left(x\right),1-I^{c}\left(x\right)\right\}$$

(4) $$H_{d}\left(x\right)=\left|I_{si}^{h}\left(x\right)-I^{h}\left(x\right)\right|$$

Here, *c* represents the R,G,B color channels, and $I_{si}^{h}\left(x\right)$ and $I^{h}\left(x\right)$ denote respectively the hue channel of the semi-inverse image and the original image in the HSV color space. Region-level hue disparity $H_{d}\left(s\right)$ is expressed as the average value of $H_{d}\left(x\right)$ in each segmentation region, as shown in Figure 3d.

f4: Color saturation

(5) $$S\left(x\right)=1-\frac{min_{c\in \left\{r,g,b\right\}}I^{c}\left(x\right)}{max_{c\in \left\{r,g,b\right\}}I^{c}\left(x\right)}$$

In hazy images, the sky regions often have lower color saturation, while the foreground objects usually appear with more abundant colors. Therefore, the color saturation feature can also provide cues for sky detection. In the HSV color space, the color saturation is defined as Equation (5). In each segmentation region, we use the average value of S(x). Region-level color saturation S(s) is shown in Figure 3e.

#### 3.2.2. Gradient Features

From Figure 3b–e we can find that the color features mentioned above cannot well express distinction between sky and some non-sky regions with sky-like color (e.g., the white horseback and the light blue sea surface). At this time, gradient features may help to identify these false sky regions.

f5: Contrast energy

Because contrast carries key information about the surface geometry and changes in the surface albedo [23], the distribution of contrast in an image is of particular importance for visual perception. Choi et al. [17] employed Contrast Energy (CE) [23] to predict the perceived local contrast on natural images. The CE is defined as Equations (6) and (7) [17,23].

(6) $$CE^{c}\left(x\right)=\frac{\alpha \cdot Z^{c}\left(x\right)}{Z^{c}\left(x\right)+\alpha \cdot k}-\tau_{c}$$

(7) $$Z^{c}\left(x\right)=\sqrt{{\left(I^{c}\left(x\right)⊗g_{h}\right)}^{2}+{\left(I^{c}\left(x\right)⊗g_{v}\right)}^{2}}$$

Here, $c\in \left\{gray,yb,rg\right\}$ is the color channels of I(x), gray=0.299R+0.587G+0.114B, yb=0.5(R+G)−B and rg=R−G. The symbol ⊗ denotes convolution, while $g_{h}$ and $g_{v}$ are the horizontal and vertical second-order derivatives of the Gaussian function, respectively; α is the maximum value of $Z^{c}\left(x\right)$; k=0.1 is the contrast gain; and $\tau_{c}=\left\{0.2353,0.2287,0.0528\right\}$ is the noise threshold in each channel. Parameters are set according to the neuronal experiments conducted in [24]. Region-level contrast energy CE(s) is expressed as the average value of CE(x) in each segmentation region, as shown in Figure 3f. We can see that the false sky regions can be identified easily by the CE. 

f6: Canny edge

In the foreground regions, objects usually exhibit some geometry shapes that can be characterized by the edges. On the contrary, sky regions have little edge information. We utilize the Canny operator to detect object edges in the foreground regions.

(8) $$E\left(x\right)=max_{c\in \left\{r,g,b\right\}}Edge^{c}\left(x\right)$$

Here, Edge${}^{c}\left(x\right)$ is the binary Canny edge of pixel *x* in channel *c*. We use the maximum value of Edge${}^{c}\left(x\right)$ in each pixel. The region-level edge feature E(s) is expressed as the average value of E(x) in each segmentation region, as shown in Figure 3g.

f7: Color Gradient

In a hazy image, color in the sky region is relatively smooth, while color in the non-sky regions is rich in changes. We employ the color gradient to measure the color variation.

(9) $$G\left(x\right)=\sum_{c\in \left\{r,g,b\right\}}\sqrt{G_{h}^{c}{\left(x\right)}^{2}+G_{v}^{c}{\left(x\right)}^{2}}$$

Here, $G_{h}^{c}\left(x\right)$ and $G_{v}^{c}\left(x\right)$ are the horizontal and vertical Sobel gradients of channel *c*. We use the cumulative value of the three channel gradients in each pixel. Region-level color gradient G(s) is expressed as the average value of G(x) in each segmentation region, as shown in Figure 3h.

#### 3.2.3. Position Features

As a matter of fact, sky usually appears in the upper part of the image, while ground usually appears in the lower part of the image. Therefore, we employ the following position features.

f8–f9: Height

(10) $$Y\left(x\right)=y\left(x\right)/H_{img}$$

Here, y(x) is the vertical height of pixel *x*, and $H_{img}$ is the height of the image. In each segmentation region, we use the maximum height value (f8) and the minimum height value (f9) to characterize the position feature, as shown in Figure 3i,j.

### 3.3. Two-Stage Sky Detection

After extracting the specific features in each sample region, the next step is to determine whether the sample belongs to sky or not. Usually, a classifier is employed to achieve this goal. However, limited to the performance of one classifier, the classification results are often unsatisfactory. To produce more accurate results, many post-processing approaches [1,14,25] have been designed to refine the sky. In this paper, we propose a two-stage scheme to detect the sky more accurately. In the first stage, we employ two SVM classifiers, a sky-concerned one and a non-sky-concerned one, to divide the input image into three subregions: high confidence sky regions $S_{sky\_hc}$, high confidence non-sky regions $S_{nsky\_hc}$ and uncertain regions $S_{uncertain}$. In the second stage, a similarity measurement is conducted to label the uncertain samples.

#### 3.3.1. Region Classification with Two SVMs

Inspired by the imbalance learning theory [26,27], we can realize that the performance of a classifier is affected by the number of training samples. The imbalance classifier is trained by the dataset in which one class has a larger number of instances than other classes. When facing the binary classification problem, most imbalance classifiers only have a good coverage for the majority class. Some special approaches were proposed to overcome this limitation. However, in this paper, the limitation is just regarded as a useful property for the design of our method. The imbalance classifier can detect almost all of the majority samples, which means that the recall of the majority class is high. Meanwhile, the precision of the minority class is high. This means that when the imbalance classifier labels a sample into minority classes, the determination will have very high credibility. This is the inspiration for designing our algorithm.

We use two SVM classifiers to determine that the candidate regions are sky or non-sky. A sky region is labeled as “positive”, and a non-sky region is labeled as “negative”. The Imbalance Level IL is defined as the ratio of the number of positive samples to that of the negative samples. Two imbalance SVMs are listed as follows:

- SVM${}_{1}$: This is a sky-concerned classifier. The positive class is the majority class in the training set, which means that the imbalance level $IL_{1}$ is greater than one.
- SVM${}_{2}$: This is a non-sky-concerned classifier. The positive class is the minority class in the training set, which means that the imbalance level $IL_{2}$ is lesser than one.

When training the SVM${}_{1}$, we use all the positive samples in the sample set, and the number of negative samples we used is half the number of positive samples ($IL_{1}=$ 2:1); when training the SVM${}_{2}$, we use all the positive samples and all the negative samples in the sample set. Actually, the numbers of positive and negative samples produced by the two segmentation methods [19,21] are different. $IL_{2}=$ 1:5 when the graph-based segmentation method [21] is used on the HazySky dataset, and $IL_{2}=$ 1:9 when the hierarchical segmentation method [19] is used on the HazySky dataset.

**Figure 4:**
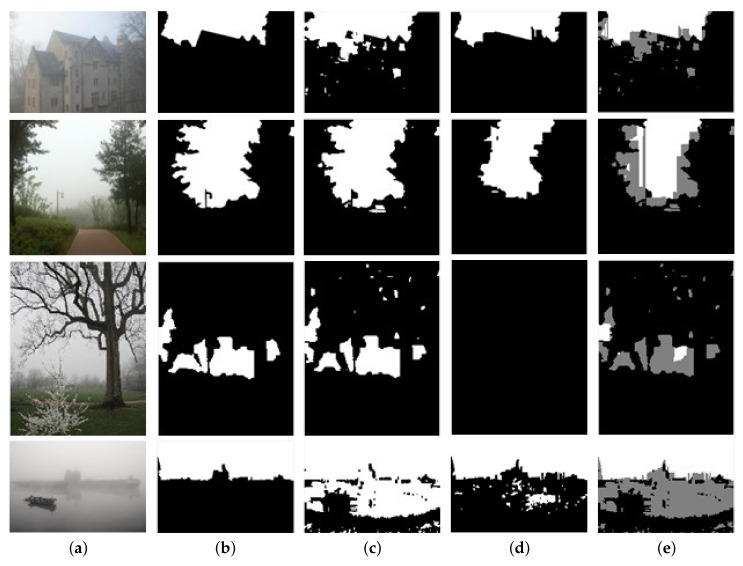
Sky detection results of the first stage. (**a**) Input hazy images; (**b**) ground truth sky; (**c**) classification results of SVM${}_{1}$; (**d**) classification results of SVM${}_{2}$; (**e**) division of the three subregions: white denotes high confidence sky regions; black denotes high confidence non-sky regions; and gray denotes uncertain regions.

After the training process, we obtained two biased classifiers. SVM${}_{1}$ has a good coverage for the sky class, but tends to misclassify some non-sky samples into the sky class. Conversely, SVM${}_{2}$ has a good coverage on the non-sky class, but tends to misclassify some sky samples into the non-sky class. Several intuitive examples of the classification results of SVM${}_{1}$ and SVM${}_{2}$ are shown in Figure 4c,d.

The precision and recall of SVM${}_{1}$ and SVM${}_{2}$ are shown in Table 1. It is obvious that, in the sky regions, SVM${}_{1}$ achieves higher recall than that of SVM${}_{2}$, while SVM${}_{2}$ achieves higher precision than that of SVM${}_{1}$. Conversely, in the non-sky regions, SVM${}_{2}$ achieves higher recall than that of SVM${}_{1}$, while SVM${}_{1}$ achieves higher precision than that of SVM${}_{2}$.

Based on the classification results of SVM${}_{1}$ and SVM${}_{2}$, we can divide the image regions into three subregions: high confidence sky regions $S_{sky\_hc}$, high confidence non-sky regions $S_{nsky\_hc}$ and uncertain regions $S_{uncertain}$. We take negative samples predicted by SVM${}_{1}$ as the $S_{nsky\_hc}$, as shown by Equation (11).

- High confidence non-sky regions $S_{nsky\_hc}$:
  (11) $$S_{nsky\_hc}=\left\{s|V_{svm_{1}}<0.5\right\}$$
- High confidence sky regions $S_{sky\_hc}$:
  (12) $$S1=\{s|V_{svm_{2}}>th\}$$
  (13) $$S_{sky\_hc}=upper\;part\;of\left\{S1\right\}$$

Here, $V_{svm_{i}}\left(s\right)$, i∈{1,2} denote the predictive values of SVM${}_{i}$ representing the sky confidence of sample *s*. Mapped by the sigmoid function, the value range of $V_{svm_{i}}\left(s\right)$ is (0,1). $V_{svm_{i}}\left(s\right)\in \left[0.5,1\right)$ indicates that *s* belongs to the sky class, and $V_{svm_{i}}\left(s\right)\in \left(0,0.5\right)$ denotes that *s* belongs to the non-sky class.

As for the $S_{sky\_hc}$, we can directly use positive samples predicted by SVM${}_{2}$ as the high confidence sky regions, as shown by Equation (12) (when th=0.5, S1 represents positive samples labeled by SVM${}_{2}$). However, from Table 1, we can see that the precision of SVM${}_{2}$ in the sky regions is still unsatisfactory. Further reducing the value of $IL_{2}$ may be a solution, but doing that will result in it being impossible for SVM${}_{2}$ to detect any sky region in some images. Therefore, to further improve the precision of SVM${}_{2}$ in the sky regions, two auxiliary criteria are added to determine the $S_{sky\_hc}$.

Firstly, as shown in the third row of Figure 4d, SVM${}_{2}$ may be too strict to identify some sky samples, so we loosen the constraint a little by setting th smaller than the border threshold (0.5). Here, we experimentally set th=0.4. Secondly, sometimes the sky regions obtained by Equation (12) may be divided into several disconnected regions in the vertical direction, as shown in the last row of Figure 4d. There exist false sky regions in the result of SVM${}_{2}$. In this case, we just take the “upper part” sky regions as the $S_{sky\_hc}$, as denoted by Equation (13). Here, the “upper part” denotes the first segment of sky in each column of the image.

Thus, we have completed the division of the three subregions, as shown in Figure 4e, where white regions denote the $S_{sky\_hc}$, black regions denote the $S_{nsky\_hc}$ and gray regions denote the $S_{uncertain}$. By adding the above two auxiliary criteria, the detection precision of SVM${}_{2}$ in the sky regions has been further improved, as shown in the Table 1. Here, we use SVM${}_{2}+$ to denote the SVM${}_{2}$ that adds the above two auxiliary criteria.

So far, when the hierarchical segmentation [19] is used, SVM${}_{1}$ can detect 89.38% of non-sky regions with a precision of 98.01%, and SVM${}_{2}+$ can detect 80.5% of sky regions with a precision of 94.65%. Furthermore, when the graph-based segmentation [21] is used, SVM${}_{1}$ can detect 87.77% of the non-sky regions with a precision of 97.86%, and SVM${}_{2}+$ can detect 79.23% of the sky regions with a precision of 93.86%. In addition, there are also some regions that are not labeled, and most of them are just the regions for which SVM${}_{1}$ and SVM${}_{2}$ produce different labels. These regions are defined as uncertain regions and will be labeled in the second stage.

#### 3.3.2. Uncertain Regions Labeling

The second stage is to label each uncertain sample as sky or non-sky. In this stage, the $S_{sky\_hc}$ and $S_{nsky\_hc}$ obtained in the first stage are taken as the reference, and a similarity measurement is conducted by computing the Euclidean distance of feature vectors between the candidate sample to the reference sky and reference non-sky regions, respectively, as shown in Equations (14) and (15).

(14) $$Dis\_p\left(s\right)=\sqrt{\sum_{i\in \left\{1,2,3,\ldots 9\right\}}\omega_{i}\cdot {\left(f_{i}\left(s\right)-\mu_{i}^{sky\_ref}\right)}^{2}}$$

(15) $$Dis\_n\left(s\right)=\sqrt{\sum_{i\in \left\{1,2,3,\ldots 9\right\}}\omega_{i}\cdot {\left(f_{i}\left(s\right)-\mu_{i}^{nsky\_ref}\right)}^{2}}$$

Here, all the features have been normalized; *s* is the index of sample; *i* is the index of feature. $\mu_{i}^{sky\_ref}$ and $\mu_{i}^{nsky\_ref}$ are the mean value of features in the sky reference and adjacent non-sky reference regions, respectively. Dis_p(s) is the distance of features between the candidate sample to the reference sky regions; Dis_n(s) is the distance of features between the candidate sample to the reference non-sky regions; and $\omega_{i}$ is the feature weight. Finally, the candidate sample will be labeled as the class with a small Euclidean distance.

Here, we use all the sky references, while only using the adjacent non-sky reference to determine the uncertain samples. That is because, in a hazy image, sky is relative smooth, and features of the sky regions are usually similar. Conversely, features of the non-sky regions in an image may vary greatly. A non-sky region may just be similar to its adjacent ones, while having a big difference from distant ones. Therefore, when labeling the uncertain regions, we constantly search for non-sky references from adjacent regions, until the total number of pixels in these non-sky reference regions reaches the preset threshold $N_{th\_adj}$. Besides, when labeling the uncertain regions, we keep the sky reference invariant, while updating the non-sky reference constantly.

### 3.4. Summary of the Whole Sky Detection Algorithm

To elaborate on the whole procedure of our sky detection approach clearly, we give the pseudocodes of our algorithm as Algorithm 1.
**Algorithm 1:** Sky detection algorithm.**1.** Segment image I(x) into small homogeneous regions, each region corresponding to a sample *s*.**2.** Compute features from $f_{1}\left(s\right)$–$f_{9}\left(s\right)$ for each sample.**3.** Classify each sample by SVM${}_{1}$ and SVM${}_{2}$, and obtain $V_{svm1}\left(s\right)$ and $V_{svm2}\left(s\right)$**4.** Detect the high confidence non-sky regions $S_{nsky\_hc}$ according to Equation (11).**for** *s* = 1 **to** max(*s*) **do** **if** Equation (11) is true **then**  $s\in S_{nsky\_hc}$ **end if****end for****5.** Detect the high confidence sky regions $S_{sky\_hc}$ according to Equation (12), Equation (13)**for** *s* = 1 **to** max(*s*) **do** **if** Equation (13) is true **then**  $s\in S_{sky\_hc}$ **end if****end for****6.** Uncertain regions’ labeling$\left\{sky\_ref\right\}=S_{sky\_hc}$$\left\{nsky\_ref\right\}=S_{nsky\_hc}${sky}=∅**for** *s* = 1 **to** max(*s*) **do** **if** (s∉{sky_ref})⋂(s∉{sky})⋂(s∉{nsky_ref}) **then**  s∈{uncertain}  Compute the Euclidean distance between sand the sky reference by Equation (14).   Compute the Euclidean distance between s and the adjacent non-sky reference by Equation (15).  **if** Dis_p<Dis_n **then**   s∈{sky}  **else**   s∈{nsky_ref}  **end if** **end if****end for**

## 4. Evaluation Metrics

To evaluate the detection accuracy of our results quantitatively, we employ the detection rate metrics [1] and the misclassification rate metric [15].

- Detection rate:
  (16) $$P_{sky}=\frac{N_{sky\_detected}}{N_{sky}}\times 100\%$$
  (17) $$P_{nsky}=\frac{N_{nsky\_detected}}{N_{nsky}}\times 100\%$$
- Misclassification rate:
  (18) $$MCR=\frac{N_{sky\_false}+N_{nsky\_false}}{N_{sky}+N_{nsky}}\times 100\%$$

Here, $N_{sky}$ is the number of pixels belonging to the ground truth sky regions, and $N_{sky\_detected}$ denotes the number of sky pixels labeled correctly. $N_{nsky}$ is the number of pixels belonging to the ground truth non-sky regions, and $N_{nsky\_detected}$ denotes the number of non-sky pixels labeled correctly. $N_{sky\_false}$ and $N_{nsky\_false}$ denote respectively the number of false sky pixels and false non-sky pixels in the detection results.

## 5. Experiments

To demonstrate the effectiveness of our proposed approach, we conduct extensive experiments on our HazySky dataset and a subset of the SkyFinder dataset [15]. On the HazySky dataset, we compare our method against three recently proposed sky detection algorithms (Shen et al.’s [1], Lu et al.’s [6] and Shang et al.’s [14]). On the SkyFinder dataset, we compare our method against the same baseline methods (Lu et al.’s [6], Hoiem et al.’s [8] and Tighe et al.’s [9]) evaluated by Mihail et al. [15]. Because Mihail et al. have reported the MCR scores of these three methods, we directly fetch the MCR values for comparison.

In the process of SVM training and predicting, we use the open source tool “Library for Support Vector Machines (LibSVM [28])” to perform SVM training and predicting tasks. LibSVM is an integrated software for support vector classification, regression and distribution estimation. It provides an easy-to-use interface where users just need to feed it with samples and labels for training and predicting. Except the parameter “h”, we set all other parameters as default values when using LibSVM (“h” is used to set the shrinking heuristics function, and we disable it by setting “h = 0”). The default kernel function is the radial basis function.

### 5.1. Training on the HazySky Dataset

We take a cross-validation strategy by dividing 500 images of the HazySky dataset into 5 splits at random (each with 100 images). In each test, we fetch 1 split of images to test and use the remaining 4 splits of images to train. When setting up the training set, we get rid of bad samples that mix sky and non-sky pixels by setting two conditions. Positive samples satisfy $\frac{N\_sky}{N}>0.8$, and negative samples satisfy $\frac{N\_nsky}{N}>0.8$, where N_sky and N_nsky denote respectively the number of sky and non-sky pixels within a sample region, and *N* denotes the total number of pixels within a sample region. For each round of training, we obtained 2 classifiers (a sky-concerned SVM and a non-sky concerned SVM). Based on the 2 SVMs, we build a sky labeling model by our two-step method mentioned in Section 3.3. After 5 rounds of training and testing, we obtain 5 sky labeling models and sky labeling results of all 500 images in the HazySky dataset.

### 5.2. Training on the SkyFinder Dataset

Because the full SkyFinder dataset cannot be available, we get 45 of the 53 cameras shared (more than 80K images). As these images were captured by static cameras, images taken by the same camera have very high similarity. In this paper, we take a subset of the SkyFinder dataset to evaluate the performance of our method. We randomly select 20% of the images from each camera, totaling about 16K images, to conduct our training and testing operation.

We take the same validation strategy as employed on the HazySky dataset by dividing these 45 cameras into 3 splits. In order to reflect the arbitrariness of the dataset division, we sort the cameras by name and divide the cameras sequentially. In each round of test, we take 1 split of images as the test set and the other 2 splits of images as the training set. In fact, our method requires only a few training samples to train. When building the training set in each round of test, we use only 10% of training samples by fetching training samples with a step length of 10 samples. After 3 rounds of training and testing, we obtained 3 sky labeling models and sky detection results of all 3 splits of the images (nearly 16K images).

### 5.3. Performance on the HazySky Dataset

The detection rate and the misclassification rate on the HazySky dataset are shown in Table 2, here, Our(G-Seg) is our results that segment the image by the graph-based method [21], and Our(H-Seg) is our results that segment the image by the hierarchical segmentation method [19]. Experimental results demonstrate that our approach achieves better performance, both on the detection rate and misclassification rate, than the other three competitors.

**Figure 5:**
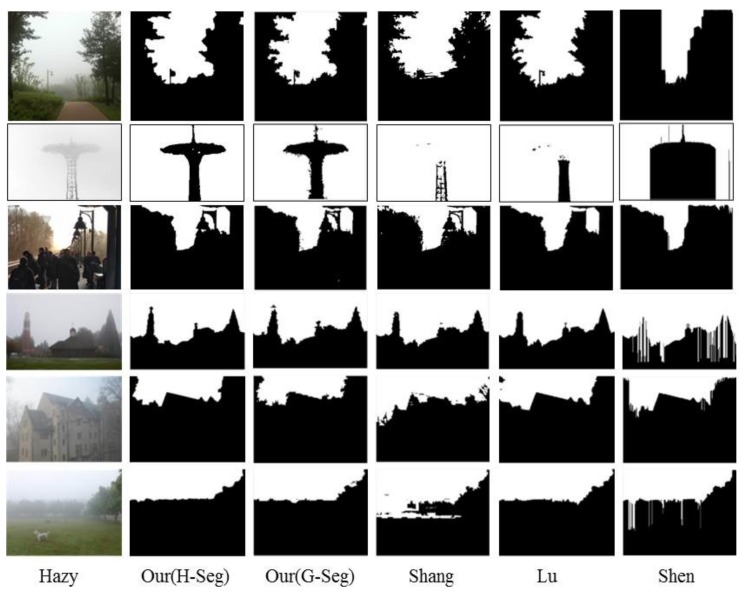
Sky detection results on the HazySky dataset. The first column is the hazy images. The second column to the last column are the results of Our(H-Seg), Our(G-Seg), Shang’s [14], Lu’s [6] and Shen’s [1] methods.

**Figure 6:**
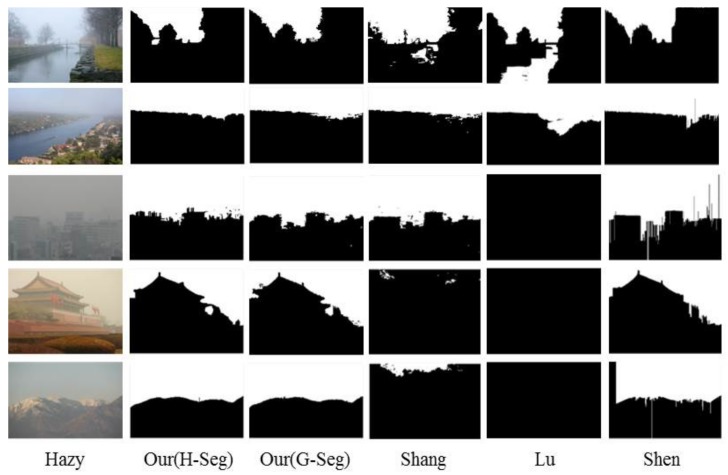
Sky detection results on the HazySky dataset. The first column is the hazy images. The second column to the last column are the results of Our(H-Seg), Our(G-Seg), Shang’s [14], Lu’s [6] and Shen’s [1] methods.

Figure 5 and Figure 6 give some examples of the sky detection results produced by these algorithms on the HazySky dataset. Shen et al.’s method [1] cannot detect sky interrupted by foreground objects in the vertical direction (as shown in the first and second row of Figure 5). Lu et al.’s method [6] produces fine edges on the detection results, but it performs badly in non-sky regions with sky-like colors (as shown in the first and second row of Figure 6). Besides, in some dense hazy scenes, Lu et al.’s method [6] is completely inefficient (as shown in the third to fifth row of Figure 6). Shang et al.’s method [14] also performs badly when the texture features and line features are weak (as shown in the fourth column of Figure 5 and Figure 6). All three algorithms [1,6,14] adapt poorly to hazy scenes, and our method produces the best results under different sky scenes of hazy images.

### 5.4. Performance on the SkyFinder Dataset

On the SkyFinder dataset, we directly fetch MCR scores of the corresponding images of the three methods (Hoiem et al.’s [8], Tighe et al.’s [9] and Lu et al.’s [6]) to compare. In the similarity measurement step, we set the feature weights experimentally as $\omega_{1:9}=\left[0.2,0.2,0.2,0,0.1,0,0,0.1,0.2\right]$ and set $N_{th\_adj}=5\%$ of the total number of pixel in the image. We report the experimental results from three aspects: time, weather and scene (camera).

**Figure 7:**
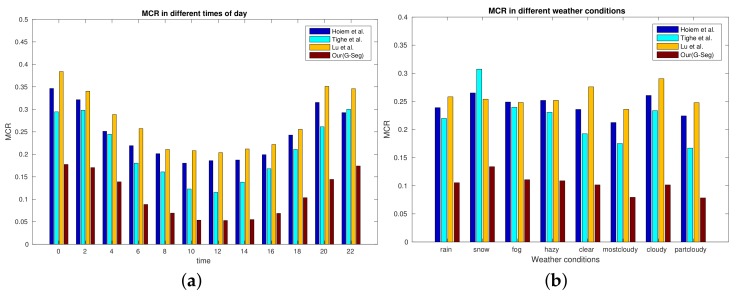
Performance analysis of our model in different weather and lighting conditions. (**a**) MCR (MisClassificationRate) values at different times of day; (**b**) MCR values in different weather conditions.

Experimental results at different times of day are shown in Figure 7a. This denotes that the lighting condition has a significant impact on the performance of sky labeling algorithms. Similar to other methods, our model achieved good detection accuracy during day time, while performing poorly for night scenes. Performances in different weather conditions are shown in Figure 7b. The results demonstrate that our model achieved better performance in all weather conditions than the other three methods.

Sample images’ test results for each camera are shown in Table 3. The results indicate that although the weather and lighting conditions have a great impact on the performance of sky labeling methods, scene content is also a key factor in determining the performance of sky labeling methods. Our method achieved an average MCR of 9.63%, which is better than the other three methods (Hoiem et al.’s 22.83%, Tighe et al.’s 19.51% and Lu et al.’s 25.08%) on our testing split. Mihail et al. [15] reported their own average MCR as 12.96% across their own testing split, which is a little worse than our result.

**Figure 8:**
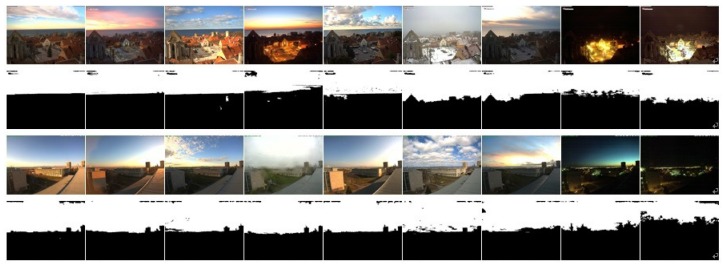

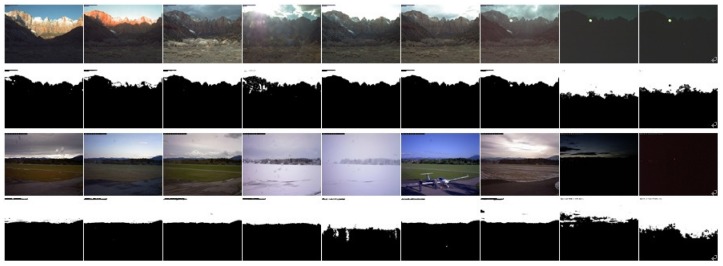
Our sky detection results on the SkyFinder dataset. We selected a number of images captured in different weather and lighting conditions from 4 cameras. The odd rows show the input images, and the even rows are the corresponding sky detection results.

Figure 8 gives some examples of the sky detection results of our method on the SkyFinder dataset. The results show that our method has a relatively strong ability to adapt to the changes of weather, time and scene. Obviously, similar to other methods, our model performs poorly in some night scenes, because night scenes weaken the features of color and gradient.

## 6. Discussion and Conclusions

In this paper, a novel algorithm for sky detection of hazy images was proposed. Different from most existing solutions, we address this problem from the perspective of probing the density of haze and characterize the sky by several haze-relevant features. Our approach can detect sky with an arbitrary shape by image segmentation and a two-stage classification. To detect sky accurately, two imbalance SVM classifiers were trained to detect the high confidence sky regions and the high confidence non-sky regions, respectively. Then, these high confidence regions were taken as the reference to further label the remaining uncertain regions. In addition, an abundant sky dataset of hazy scenes was built for model training and performance evaluation of the proposed sky detection algorithm.

Performance evaluation was conducted both on our HazySky dataset and the SkyFinder dataset. Experimental results on the HazySky dataset demonstrated that our sky detection method uses fewer features while achieving better performance both on the detection rate and the misclassification rate in hazy images. Experimental results on the SkyFinder dataset demonstrated that our method achieved better performance not only in hazy scenes but also in other weather conditions (e.g., rain, snow, cloudy and clear) and also adapts well to the changes of daytime lighting conditions. Besides, unlike the convolution neural network-based methods, our method is more flexible, easier to train (it needs only few training samples) and more suitable for embedded system applications.

In spite of this, we still insist that existing hand engineered feature-based methods remain effective, so long as they are modified according to the task’s need (e.g., add some other features, or retrain their model in the specific application environment). In the future, we will study the characteristics of sky under different objective conditions, analyze the effectiveness of sky-relevant features in different application environments and explore more powerful sky detection methods by adding environment priors (e.g., weather, time, season, etc.). 

## Figures and Tables

**Table 1:** Recall and precision of SVM${}_{1}$, SVM${}_{2}$ and SVM${}_{2}+$.

		H-Seg			G-Seg		
		SVM${}_{1}$	SVM${}_{2}$	SVM${}_{2}+$	SVM${}_{1}$	SVM${}_{2}$	SVM${}_{2}+$
**Recall**	$R_{sky}$	0.9575	0.8338	0.8050	0.9524	0.8734	0.7923
	$R_{nsky}$	0.8938	0.9762	0.9840	0.8777	0.9658	0.9828
**Precision**	$P_{sky}$	0.7939	0.9136	0.9465	0.7580	0.8608	0.9386
	$P_{nsky}$	0.9801	0.9511	0.9348	0.9786	0.9692	0.9345

**Table 2:** Detection rate and misclassification rate on the HazySky dataset. In the similarity measurement step, we set the feature weights experimentally as $\omega_{1:9}=\left[0.3,0.4,0,0,0.2,0,0,0,0.1\right]$ and set $N_{th\_adj}=1\%$ of the total number of pixel in the image.

	Shen [1]	Lu [6]	Shang [14]	Our(G-Seg)	Our(H-Seg)
$P_{sky}\left(\%\right)$	80.14	88.99	89.70	92.26	92.89
$P_{nsky}\left(\%\right)$	93.31	94.88	93.77	95.89	96.60
MCR(%)	10.05	6.62	7.57	5.04	4.35

**Table 3:** Test results of 20% sample images for each camera in the SkyFinder dataset (%). We report the detection rate and misclassification rate of our method in each camera, as well as the average misclassification rate of the four methods.

Split 1				Split 2				Split 3			
**Camera**	$P_{\mathrm{sky}}$	$P_{\mathrm{nsky}}$	**MCR**	**Camera**	$P_{\mathrm{sky}}$	$P_{\mathrm{nsky}}$	**MCR**	**Camera**	$P_{\mathrm{sky}}$	$P_{\mathrm{nsky}}$	**MCR**
**10066**	93.88	89.85	8.15	**3395**	85.26	96.72	9.63	**684**	91.97	97.41	6.00
**10870**	99.15	86.79	9.94	**3396**	89.02	89.42	10.78	**7211**	98.22	96.10	3.11
**10917**	-	82.01	17.99	**3837**	96.16	84.08	13.55	**7233**	98.05	91.77	7.28
**1093**	97.50	94.71	4.49	**3888**	99.09	80.78	15.61	**7371**	98.21	85.61	8.52
**11160**	93.90	96.68	4.57	**4181**	95.06	88.27	10.03	**75**	81 39	98.57	15.60
**11331**	73.12	94.59	10.40	**4232**	90.49	95.57	7.22	**8438**	67.05	97.59	14.58
**162**	93.57	99.32	4.67	**4584**	86.65	97.84	7.30	**858**	86.30	99.71	7.56
**17218**	62.96	95.88	19.84	**4679**	89.83	90.91	9.35	**861**	98.87	80.60	13.93
**17244**	92.11	95.65	5.16	**4795**	98.64	81.79	17.80	**8733**	83.80	99.00	10.55
**19106**	93.43	97.83	4.37	**4801**	94.37	97.98	3.66	**8953**	88.07	84.11	13.72
**19306**	97.82	87.60	8.88	**5020**	95.14	98.39	3.11	**9112**	89.34	95.82	6.88
**19388**	98.62	90.53	6.31	**5021**	92.94	98.00	4.37	**9291**	98.15	86.28	11.39
**204**	84.03	82.49	16.61	**623**	90.95	98.91	5.78	**9483**	98.17	96.18	3.02
**260**	99.01	91.02	5.98	**65**	59.85	99.37	22.23	**9708**	96.68	88.52	10.75
**3297**	73.15	95.70	15.33	**6798**	81.50	96.09	12.63	**9730**	96.20	96.45	3.63
**Average MCR w.r.t Camera**				**Hoiem** [8]		**Tighe** [9]		**Lu** [6]		**Ours**	
				22.83		19.51		25.08		9.63	
